# No evidence of sexual dimorphism in the tails of the swallowtail butterflies *Papilio machaon gorganus* and *P. m. britannicus*

**DOI:** 10.1002/ece3.7374

**Published:** 2021-03-17

**Authors:** Lydia K. Koutrouditsou, Robert L. Nudds

**Affiliations:** ^1^ School of Biological Sciences, Faculty of Biology, Medicine and Health University of Manchester Manchester UK

**Keywords:** butterfly morphology, Lepidoptera, predation, sexual dimorphism, sexual selection, wings

## Abstract

The European swallowtail butterfly (*Papilio machaon*) is so named, because of the long and narrow prominences extending from the trailing edge of their hindwings and, although not a true tail, they are referred to as such. Despite being a defining feature, an unequivocal function for the tails is yet to be determined, with predator avoidance (diverting an attack from the rest of the body), and enhancement of aerodynamic performance suggested. The swallowtail, however, is sexually size dimorphic with females larger than males, but whether the tail is also sexually dimorphic is unknown. Here, museum specimens were used to determine whether sexual selection has played a role in the evolution of the swallowtail butterfly tails in a similar way to that seen in the tail streamers of the barn swallow (*Hirundo rustica*), where the males have longer streamers than those of the females. Previously identified sexual dimorphism in swallowtail butterfly size was replicated, but no evidence for dimorphism in tail length was found. If evolved to mimic antennae and a head to divert a predatory attack, and if an absolute tail size was the most effective for this, then the tail would probably be invariant with butterfly hindwing size. The slope of the relationship between tail length and size, however, although close to zero, was nonetheless statistically significantly above (tail length ∝ hindwing area ^0.107 ± 0.011^). The slope also did not equate to that expected for geometric similarity (tail length ∝ hindwing area^1/2^) suggesting that tail morphology is not solely driven by aerodynamics. It seems likely then, that tail morphology is primarily determined by, and perhaps a compromise of several, factors associated with predator avoidance (e.g. false head mimicry and a startling function). Of course, experimental data are required to confirm this.

## Graphical abstract

Despite being the defining feature of the swallowtail butterfly (*Papilio machaon*), an unequivocal function for the tail is yet to be identified. We can now, however, conclude that they are unlikely to be the result of sexual selection.

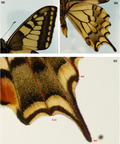

## 1 INTRODUCTION

Sexual selection is responsible for some of the most ornate traits seen in animals (Darwin, 1871; Zahavi, 1975). A common characteristic among many species of the Papilionidae family is the pair of narrow protuberances on the trailing edge of their hindwings. Although part of the wing, they are usually referred to as tails and give the Papilionidae their common name (swallowtail), but their function(s) has yet to be unequivocally determined (Collins & Morris, 1985). Elongated tails in some birds are the product of sexual selection conferring a reproductive advantage to males possessing longer feathers (Andersson, 1982; Møller, 1988).

The question of whether the swallowtail butterfly tail is sexually dimorphic in size and potentially the result of sexual selection (Darwin, 1871; Zahavi, 1975), however, has not been addressed. Elongated tails in several species of bird are often driven by inter sexual selection. For example, a longer tail in male long‐tailed widowbirds (*Euplectes progne*), increases mating success by attracting more females (Andersson, 1982). Indeed, female choice appears to have elongated the tails of males in several species with both graduated and pintails (Balmford et al., 1993). In these bird examples sexual dimorphism is apparent, since females have short tails, and long tails are a male characteristic, indicating a role for sexual selection in their evolution. In contrast, both sexes in the barn swallow (*Hirundo rustica*) possess tail streamers (the outermost tail feathers are elongated). Those of the male, however, relative to the size of the bird are longer than those of the females and males with longer streamers achieve higher reproductive success (Møller, 1988).

The function of similar tails in other species of butterfly, for example the hairstreak (*Calycopis cecrops*), appear to be an anti‐predator mechanism, acting as the antennae part of a ‘false‐head’, successfully diverting an attack to the rear part of their hindwings, allowing the butterfly to escape (Sourakov, 2013). *Papilio machaon*, however, have wider club shaped tails less resembling antennae, or heads. Furthermore, swallowtails often perch with their wings open in what resembles a gliding posture (forewings pulled backwards, and hindwings close together with their rear edges above the abdomen) and false heads in other butterfly families (for example members of the family *Lycaenidae*) are created when the wings are folded together during perching (Novelo Galicia et al., 2019). A proposed predatory avoidance strategy of *Papilio machaon* is a deimatic behavior in which the butterfly rapidly flicks its wings open exposing its brightly colored dorsal wing surface. If the predation event continues it will open and close its wings in a jerky motion (Olofsson et al., 2012). In many species, butterfly wing coloration and patterning itself can play a role in both mate selection and predator avoidance (Nijhout, 1991), with dorsal characters playing more of a role in mate choice and the ventral in predator avoidance (Oliver et al., 2009). Whether the swallowtail's tail acts as a supernormal stimulus and plays a role in the startling of the predator is not clear.

One cost of long tail ornaments in birds is an increase in aerodynamic drag during flight (Thomas, 1997). Although the tails of the swallowtail butterflies also increase drag, they may additionally enhance lift with a resulting improvement in longitudinal static stability, at the cost of manoeuvrability (Park et al., 2010). An aerodynamic effect, however, does not preclude a role for sexual selection in the evolution of the tail. It is thought that the streamers of the barn swallow confer an aerodynamic advantage up to a certain length (that of the females). Beyond that, further elongation (seen in the males) reduces aerodynamic performance (is costly) and is a sexually selected trait driven by female choice (Nudds & Spencer, 2004; Rowe et al., 2001). The mating system of *Papilio machaon* subspp. ‐ male swallowtails hold territories and intercept any adult butterfly encroaching (Dempster et al., 1976) and a courtship involving soaring at height (Newland et al., 2015) ‐ however, would suggest that the tail, if sexually selected, would more likely be the result of intrasexual selection. Males spend much of the day in flight using a flap and glide flight style (Newland et al., 2015). Hence, the male tail could be selected for different flight requirements (aerodynamic optima) to that of the female and hence sized differently. Specifically, sexual dimorphism in the tail could be driven by male–male competition for territories and mates with the tail providing the ability to both intercept encroaching males and successfully intercept females for mating.

There are at least 37 recognized subspecies of *Papilio machaon* (Collins & Morris, 1985), of which we studied the British subsp. *Papilio machaon britannicus* (Seitz, 1907), only resident in the Norfolk area (Collins et al., 2020; Van Swaay et al., 2010), and the European mainland (henceforth just referred to as European) subsp. *Papilio machaon gorganus* (Fruhstorfer, 1922). *P. m*. *britannicus* differs from the European subsp. in color, being generally paler, in size, being smaller, type of habitat it occupies, and its larval food plant (Dempster et al., 1976). *Papilio machaon* species are sexually dimorphic. Typically, females have larger wings and associated body parts than males, in order for them to carry hundreds of eggs in their abdomen, which adds extra weight (Collins & Morris, 1985; Dempster et al., 1976).

Although a role in predator avoidance cannot be discounted, the fact that the swallowtails main defence to a predation event appears to be wing flicking behavior and the butterflies are sexually dimorphic with the males holding territories, also means that a sexually selected component to the tail's evolution cannot be discounted either. Accordingly, here the tail lengths of European and British swallowtails were measured from museum specimens. If selected for a different aerodynamic optimum and driven by intrasexual selection or the result of intersexual selection, then the tails of males would be of different length to those of females, either longer or shorter. The prediction tested here was that males would have different length tails to that of the females.

## 2 MATERIAL AND METHODS

### 2.1 Photographing the specimens

The swallowtail specimens used in the study are housed in the entomological collections of the University of Manchester Museum, the Liverpool World Museum and the Bolton Library and Museum. Photographs of the specimens were taken in the museums, using a Nikon D100 camera fitted with a Nikkor 60 mm micro lens. To avoid researcher bias, all photographs and scale bar positioning was carried out by the same researcher (RLN). The camera was mounted on an adjustable copying stand so that the lens plane was parallel to the surface of the wings being photographed. The specimens were pinned to a piece of Blu‐Tack® directly underneath the camera and on top of a lightbox (RS Components A4 LED lightbox). Additional LED light sources were used for photographs taken of the ventral view, but not for dorsal photographs. This ensured that the wing veins were visible. Scale bars were placed next to and at the same height as the wing being photographed. The fore‐ and hind‐wings of the specimens overlapped so two photographs were taken of each. One photograph of the dorsal side to show the forewing and one of the ventral side to show the hindwing. At the cessation of each photographing session, a scale bar was placed upon the light box four times: from corner to corner of the photographic frame twice; vertical from the midpoint of the top and bottom edge of the photographic frame; and horizontal from the midpoint of the left, and right sides of the photographic frame. The number of pixels within the first cm of each line's bisection with the photographic frame edge were calculated (so eight data points for each of the four photographic sessions). A two‐way ANOVA showed that although the pixels per cm differed among photographing sessions [*F*_3,21_ = 62.45, *r* ^2^ = 0.88, *p* < .001], due to slightly different lighting conditions and copy stands, they did not differ among the eight measurement points [*F*_7,21_ = 0.506, *r* ^2^ = 0.02, *p* = .820] indicating that the camera lens was always parallel with the surface of the light table.

The specimens were chosen based on the subspecies and the location at which they were collected (Britain or Continental Europe). In each drawer every specimen that was in good condition was photographed. Specimens that would not provide data for all three morphological variables (hindwing area, forewing area and tail length) due to damage were excluded. Because the specimens came from different collectors, different areas, and collection dates, the data can be considered random for each species. Specimens were sexed according to their abdomen shape and genitalia: males are characterized by a narrow more pointed abdomen with claspers, whereas claspers are absent in the females and the abdomens broader.

### 2.2 Size analyses

**FIGURE 1:**
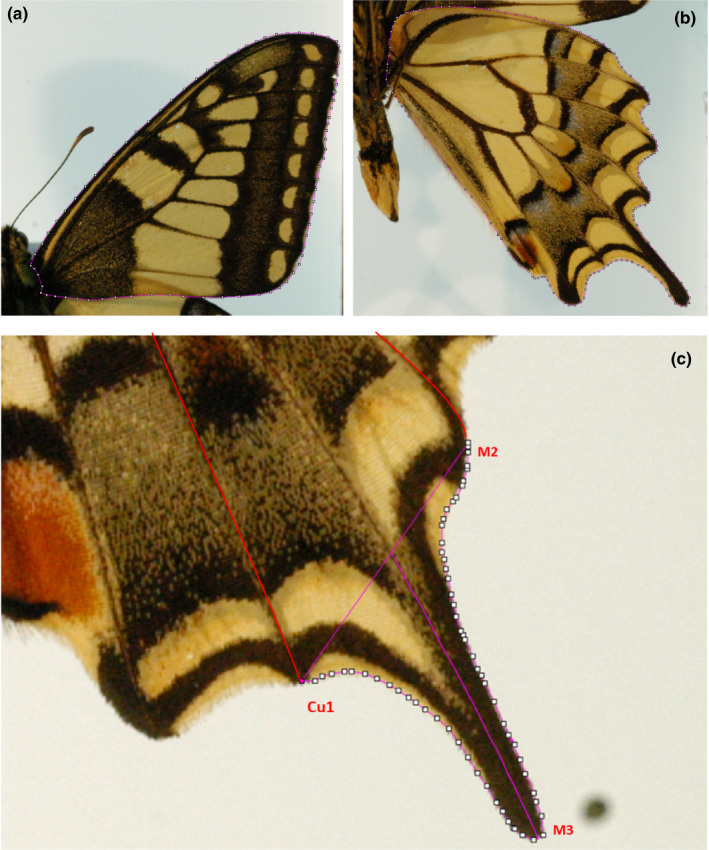
Showing the methods for measuring the wings and tail. The forewing was measured from the dorsal side (a) and the hindwing from the ventral side (b) as one wing is occluded if measured from the same side. The outline of the wings for the purposes of determining wing area are depicted by white dots joined by a red line. Tail length was measured (c) as the distance from the base (a line joining Cu1 and M2 where each meets the wing margin) to the tip along the M3 vein corrected for out of plane curvature using Equation (1). The vein nomenclature was obtained from Patil and Magdum (2017)

Wing areas were measured as size metrics, because the size of the thorax and abdomen is not an accurate representation of overall size, since the bodies of the specimens in the collections tend to curl up. All image analyses were completed by one researcher (LKK) so as not to introduce bias into the data. To ensure the wings were not occluded by the other, for each specimen the area of one forewing on the dorsal side (Figure 1a) and one hindwing on the ventral side (Figure 1b) were measured. The tail area (Figure 1c) was calculated using the “polygon selections” tool of ImageJ 1.x software (Schneider et al., 2012) and subtracted from the hindwing area, that is, hindwing area does not include tail area (Figure 1c).

Tail length was determined as follows. The points where the Cu1 and M2 (Patil & Magdum, 2017) veins met the edge of the wing were identified and a straight line was drawn between them (Figure 1c). The resulting line was used to set the base of the tail and tail length was determined as the distance from this base to the tail tip along the M3 vein calculated using the “Bezier tool” in ImageJ (Schneider et al., 2012), which is able to accommodate 2D curvature in the vein.

In many of the specimens, the tails were curled upward or downward (out of the 2D camera lens plane). During photographing the difference in height (*β*) between the tail base and the tail tip (*α*) was measured using a rule. To correct the length measurements an approximate elliptical curve was fitted to *α* and *β* measurements and the final tail length (*L*) was calculated as the ¼ of an ellipse circumference using:

(1) $$L\approx \left(2\pi \cdot \sqrt{\frac{\alpha^{2}+\beta^{2}}{2}}\right)/4$$

The mean (± standard deviation) corrected tail length was 0.96 ± 0.12 cm, which was on average 12% (0.10 ± 0.07 cm) higher than the original measured tail length. No size bias in the number of individual butterflies requiring a tail length correction was evident. Forty‐six swallowtail butterflies did not require the correction and there was no relationship (Pearson's product moment correlation = 0.09, *p* = .258) between the percentage tail length correction and butterfly size (hindwing area) for the remaining 148.

General linear models (GLM) were used (Fox & Weisberg, 2019) to test whether relative forewing wing and relative tail size differed between the sexes and subspecies. Hindwing area was included as a covariate to control for size.

Two‐way ANOVAs were used to determine whether absolute hind‐ and forewing areas differed between sexes or subspecies. The residuals from all statistical models were confirmed as approximating a normal distribution using Shapiro–Wilk tests. All the statistical analyses on size variables were conducted in R (R Core Team, 2019) using R studio (R Studio Team, 2015)

## 3 RESULTS

**FIGURE 2:**
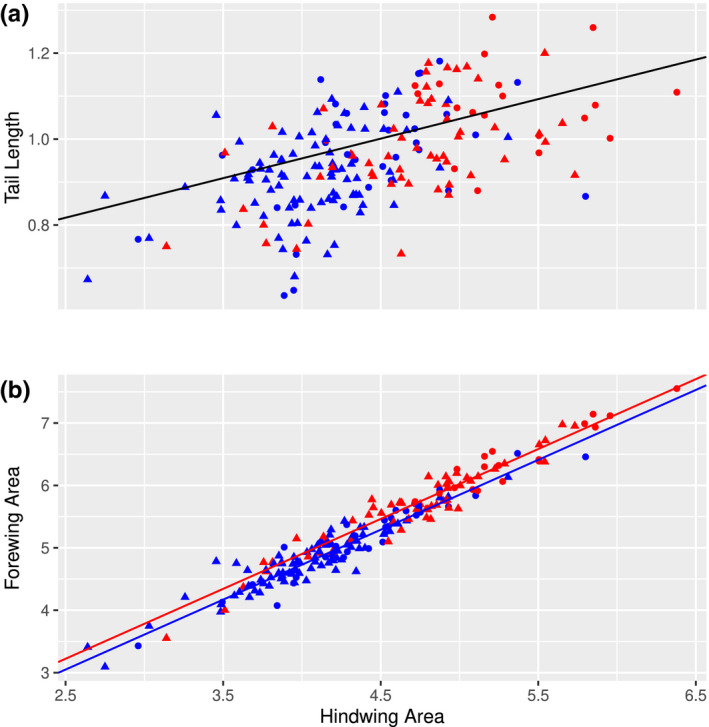
Scatter plots showing (a) tail length (cm) and (b) forewing area (cm^2^) plotted against hindwing area (cm^2^) for both sexes (females = red and males = blue) and subspecies (European = circles and British = triangles). No differences in tail length were detected between either sex or subspecies. The line of best fit showing the relationship between tail length and hindwing area is *y* = 0.486 + 0.107 ^± 0.011^ *x*. For a given hindwing area the forewing area of the females was greater than that of the males. The lines of best fit are described by *y* = 0.392 + 1.122 ^± 0.025^ *x* and *y* = 0.257 + 1.122 ^± 0.025^ *x* for the females (red line) and males (blue line) respectively

Tail length (Figure 2a) did not differ between the sexes [*F*_1,189_ = 0.65, *r* ^2^ < 0.01, *p* = .422] nor between subspecies [*F*_1,189_ = 3.08, *r* ^2^ < 0.01, *p* = .081]. The finding of no difference in tail length between sexes was also consistent between the subspecies [*F*_1,189_ = 0.36, *r* ^2^ < 0.01, *p* = .547]. Tail length increased concomitantly with increasing hindwing area [*F*_1,189_ = 38.98, *r* ^2^ = 0.17, *p* < .001].

Forewing area (Figure 2b) increased with increasing hindwing area [*F*_1,189_ = 1,642.14, *r* ^2^ = 0.89, *p* < .001]. For a given hindwing area forewing area (and *vice versa*) did not differ between the two subspecies [*F*_1,189_ = 0.01, *r* ^2^ < 0.01, *p* = .945], but was greater across all hindwing sizes in the females than in the males [*F*_1,189_ = 16.27, *r* ^2^ < 0.01, *p* < .001]. For both subspecies the females had a greater hind wing area than the males [*F*_1,189_ = 0.71, *r* ^2^ < 0.01, *p* = .400].

The females [*F*_1,190_ = 109.51, *r* ^2^ = 0.33, *p* < .001] and the European subspecies [*F*_1,190_ = 32.50, *r* ^2^ = 0.10, *p* < .001] had a greater absolute forewing area than the males and the British subspecies respectively. The between sexes difference in forewing area was consistent within each subspecies [F1,190 = 2.641, r2 < 0.01, p = .106]. The same pattern was apparent for absolute hindwing area [sex x subspecies; *F*_1,190_ = 2.039, *r* ^2^ < 0.01, *p* = .155: sex; *F*_1,190_ = 87.62, *r* ^2^ = 0.28, *p* < .001: subspecies; *F*_1,190_ = 36.39, *r* ^2^ = 0.12, *p* < .001]. The female European swallowtails had the largest wing areas, and the male British swallowtails had the smallest wing areas overall (Table 1).

**TABLE 1:** The mean (± standard deviation) forewing and hindwing areas (cm^2^) per subspecies and sex

	European females (n = 20)	British females (n = 55)	European males (n = 36)	British males (n = 83)
Mean forewing area	6.33 ± 0.59	5.61 ± 0.63	5.20 ± 0.66	4.79 ± 0.47
Mean hindwing area	5.27 ± 0.51	4.66 ± 0.52	4.42 ± 0.54	4.03 ± 0.42

## 4 DISCUSSION

There was no detectable sexual dimorphism in tail length, suggesting that sexual selection does not play a role in swallowtail tail evolution. The previously documented size dimorphism (larger females than males) and size differences between subspecies, with the European larger than the British (Collins & Morris, 1985; Dempster et al., 1976) was recovered in this dataset.

In both males and females, forewing area increased with hindwing area at a slightly higher rate (∝ hindwing area^1.122 ± 0.025^) than that expected for geometric similarity (forewing area ∝ hindwing area^1^) suggesting relatively greater forewing areas in individuals with greater hindwing areas. The slope (0.107 ± 0.011) of the relationship between tail length and hindwing area indicated an even further deviation from geometric similarity (tail length ∝ hindwing area^1/2^), with larger individuals having relatively shorter tails. The corrections made for the curvature of the tail could have introduced some error into the tail lengths. This error, however, was not size dependent and therefore should not affect the scaling of tail length with hindwing area. For a similar aerodynamic impact across the size range of the swallowtails in this current study, geometric scaling between tail length and hindwing area would be expected. Hence, the deviation from geometric similarity found implies that the aerodynamic impact of the tail differs with size and suggests a flight performance or energy cost gradient across the size range of swallowtail butterflies, with the point of lowest aerodynamic cost not known. Although it is not clear what would be expected for tail length if it functioned as a predator avoidance mechanism, perhaps if mimicking antennae and a head to direct a predatory attack to the tail rather than to the head end of the butterfly, and if a predator was selecting an absolute tail size, the tail might be expected to be invariant with butterfly hindwing size. As already intimated, however, the scaling of tail length to hindwing area was close to zero (0.107 ± 0.011) but nonetheless, statistically significantly higher than zero.

The swallowtail butterfly may employ a suite of antipredator strategies, for example, a wing flicking startling behavior (Olofsson et al., 2012) enhanced by the peripheral wing spots, which have been shown to intimidate birds in other butterfly species (Vallin et al., 2005), and a false head for directing beak strikes to the less vulnerable parts of the butterfly (Novelo Galicia et al., 2019). The tail could contribute to either of these charades. The optimum tail length for either strategy in isolation may differ and so the length evolved is a compromise between the two. This may be why tail length scales against hindwing well below what would be expected for geometric similarity. Although experimental confirmation is required, the process of elimination leads to the conclusion that the evolution of the tail is primarily driven by predator avoidance. In contrast, sexual selection as the driver of swallowtail morphology appears unlikely as does aerodynamic performance alone.

## 5 CONCLUSION

The tail of the *Papilio machaon* and *Papilio machaon gorganus* is not sexually dimorphic and hence unlikely the product of sexual selection. Similarly, the scaling of tail length with hindwing area, would suggest that aerodynamics alone has not driven the evolution of the tail. Consequently, the tail is likely to play a role in predator avoidance. Of course, the data are correlative. To confirm a role in predator or predation avoidance would require experimental data using perhaps a similar approach to that of Olofsson et al. (2012), but with butterflies with and without tails.

## CONFLICT OF INTEREST

The authors have no conflicts of interest to declare.

## AUTHOR CONTRIBUTIONS

**Lydia K. Koutrouditsou:** Conceptualization (supporting); data curation (equal); formal analysis (equal); investigation (equal); methodology (supporting); validation (equal); visualization (equal); writing‐original draft (lead); writing‐review & editing (supporting). **Robert L. Nudds:** Conceptualization (lead); data curation (equal); formal analysis (equal); investigation (equal); methodology (lead); resources (lead); supervision (lead); validation (lead); visualization (equal); writing‐original draft (supporting); writing‐review & editing (lead).

## Data Availability

The data used in this study are available for download from Dryad at https://doi.org/10.5061/dryad.ffbg79ctn.
